# Magnetic Retraction of Bowel by Intraluminal Injectable Cyanoacrylate-Based Magnetic Glue

**DOI:** 10.1155/2013/526512

**Published:** 2013-11-12

**Authors:** Zhigang Wang, Andrew Brown, Pascal André, Stuart I. Brown, Gordon J. Florence, Alfred Cuschieri

**Affiliations:** ^1^Institute for Medical Science and Technology (IMSaT), Wilson House, University of Dundee, Dundee DD2 1FD, UK; ^2^School of Physics and Astronomy, University of St Andrews, St Andrews KY16 9SS, UK; ^3^School of Chemistry, University of St Andrews, St Andrews KY16 9ST, UK

## Abstract

Magnetic retraction offers advantages over physical retraction by graspers because of reduced tissue trauma. The objectives of this study are to investigate a novel method of magnetisation of bowel segments by intraluminal injection of magnetic glue and to demonstrate the feasibility of magnetic retraction of bowel with sufficient force during minimal access surgery. Following an initial materials characterisation study, selected microparticles of stainless steel (SS410-**μ**Ps) were mixed with chosen cyanoacrylate glue (Loctite 4014). During intraluminal injection of the magnetic glue using *ex vivo* porcine colonic segments, a magnetic probe placed at the injected site ensured that the SS410-**μ**Ps aggregated during glue polymerisation to form an intraluminal mucosally adherent coagulum. The magnetised colonic segments were retracted by magnetic probes (5 and 10 mm) placed external to the bowel wall. A tensiometer was used to record the retraction force. With an injected volume of 2 mL in a particle concentration of 1 g/mL, this technique produced maximal magnetic retraction forces of 2.24 ± 0.23 N and 5.11 ± 0.34 N (*n* = 20), with use of 5 and 10 mm probes, respectively. The results indicate that the formation of an intraluminal coagulum based on SS410-**μ**Ps and Loctite 4014 produces sufficient magnetic retraction for bowel retraction.

## 1. Introduction

Retraction of bowel loops during minimal access surgery (MAS) remains problematic because the low friction slippery smooth and moist surface resists grasping. For this reason, to provide effective traction, the jaws of laparoscopic graspers feature ridges or toothed profiles to minimize slip and for this reason, the bowel is often grasped too tightly, increasing risk of trauma and associated complications, for example, delayed healing, adhesion formation, and even perforation [1]. The potential bowel trauma during laparoscopic grasping is well documented. One study [2] confirmed the low incidence of successful grasping (62%) and highlighted the need for improvements in laparoscopic grasping. This is particularly pertinent to natural-orifice transluminal endoscopic surgery (NOTES) and single port or port laparoscopic surgery (SPLS). Thus exposure of the gallbladder [3] is difficult during SPL cholecystectomy. Ryou and Thompson [4] described the use of internal and external magnets for liver retraction during experimental transcolonic (NOTES) cholecystectomy and found that the magnetic system provided effective liver retraction and significantly shortened the procedure time. 

 Magnetic interactions and magnetic force have attracted considerable research for both medical and surgical applications [5, 6]. Magnetic microparticles and small magnets have been used for MAS applications such as in magnetic tissue retraction [4, 7, 8], magnetized islets separation for transplantation [9], magnetic navigation of catheters [10] or untethered devices (e.g., microrobots or magnetic capsules) [11–13], magnetic detection and marker [14–16], and magnetic compression anastomosis [17, 18]. Magnetic fields generated by magnetic particles [14] or implanted magnets [16] have been used for the detection of tumor or lesion sites. Clinically the strong magnetic interaction between paired magnets has been used to create a compression anastomosis for revision of bilioenteric anastomotic stricture in a patient after live-related hepatic transplantation [17] and to create a choledochojejunal anastomosis [18]. 

We have been investigating tissue magnetization by magnetic nano/microparticles for MAS applications and have previously reported two tissue ferromagnetisation approaches for retraction: (i) surface magnetization by applying a small volume of glue-based magnetic media to the mucosal surface [7] and (ii) by interstitial injection of phosphate-buffered saline (PBS) ferrofluids [8]. In these experiments injected ferromagnetisation was shown to be superior to magnetisation by surface magnetic pellets as the latter tended to peel off the tissue during retraction by magnetic probes.

In the present study, we report a third novel approach for the magnetization of bowel loops for magnetic retraction during MAS and open surgery. This is based on intraluminal injection of glue-based magnetic glue which bonds on polymerization to the mucosal layer of the bowel wall. A magnetic probe is then inserted intraperitoneally and placed on the serosal aspect to retract the magnetized bowel. This paper reports the development and characterization of cyanoacrylate magnetic glues containing dispersed stainless steel microparticles.

## 2. Methods

### 2.1. Glues and Maximal Temperatures during Polymerisation

**Figure 1:**
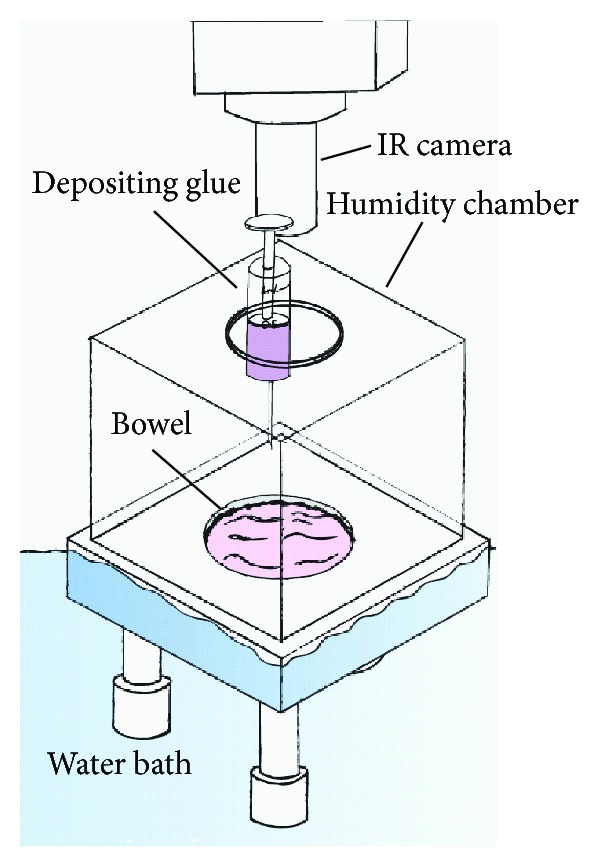
Setup for study of heat generation during polymerisation of the glues in an open bowel segment within a closed chamber.

Four medical grade glues were investigated for their suitability in the formulation of injectable magnetic glues: Dermabond (Ethicon, Somerville, NJ, USA), Indermil (US Surgical, Norwalk, NJ, USA), and Histoacryl (Braun, Melsungen, Germany) and Loctite 4014 (Henkel, Dusseldorf, Germany). All are topical skin adhesives made of octyl-cyanoacrylate or butyl-cyanoacrylate glues [19]. Since temperatures of 42°C and above are known to damage tissues [20], the extent of maximal heat generation during glue polymerisation was investigated. The heat sink effect resulting from perfusion of the bowel wall was simulated by circulation of isotonic solution by means of a pump on the serosal surface of the colon segments which were placed in a water bath.  Figure 1 illustrates a thermal camera (Cedip Jade camera, FLIR Systems, France) temperature measurement system in an open bowel experiment within a humidity chamber. In this initial study 0.5 mL of glue was deposited onto the surface of the bowel segment which was kept moist at 37°C with Hartmann's solution circulating at 7 L/min to simulate *in vivo* conditions including the heat sink of vascular perfusion. 

### 2.2. Characterization of Magnetic Microparticles

Two types of stainless steel microparticles (SS-*μ*Ps) were examined in the initial materials characterisation: 410 microparticles (SS410-*μ*Ps, Goodfellow Cambridge Ltd., Huntingdon, UK) and 430 microparticles (SS430-*μ*Ps, from Alfa Aesar, A Johnson Matthey Company, Lancashire, UK). Both materials had been previously reported in the literature [15, 21, 22] for use in medical/surgical applications. The magnetic properties of the two materials were quantified with a Superconducting Quantum Interference Device (SQUID) magnetometer (MPMS XL from Quantum Design, now part of Lot-Oriel GmbH & Co. KG). The particle crystallinity was confirmed by X-ray diffraction (XRD; STADI/P powder diffractometer from Stoe) and their sizes were determined by scanning electron microscopy (SEM; S-4800 from Hitachi). Chemical composition of all particles was also analysed by inductively coupled plasma optical emission spectrometry (ICP-OES; Optima 5300 DV from Perkin Elmer). 

### 2.3. Cyanoacrylate-Based Magnetic Glue

Loctite 4014 was chosen to formulate the injectable cyanoacrylate-based magnetic glue in view of its lower cost ease of use (single component) and its quick cure rate in high humidity conditions. Due to its very low viscosity, a range of concentrations can be mixed with magnetic particles for different applications. Concentrations of particles ranging from 0.5 to 1 g/mL were used for the magnetic bowel retraction studies. In the formulation of high concentration magnetic glue (1 g/mL magnetic fluid), 2.0 grams of SS410 magnetic particles was placed in a Sure/Seal bottle and 2 mL Loctite 4014 was added thereafter. The magnetic particles were then mixed and suspended in the liquid glue by vigorous hand shaking of the sealed bottle. 

### 2.4. Magnetic Bowel Retraction

**Figure 2:**
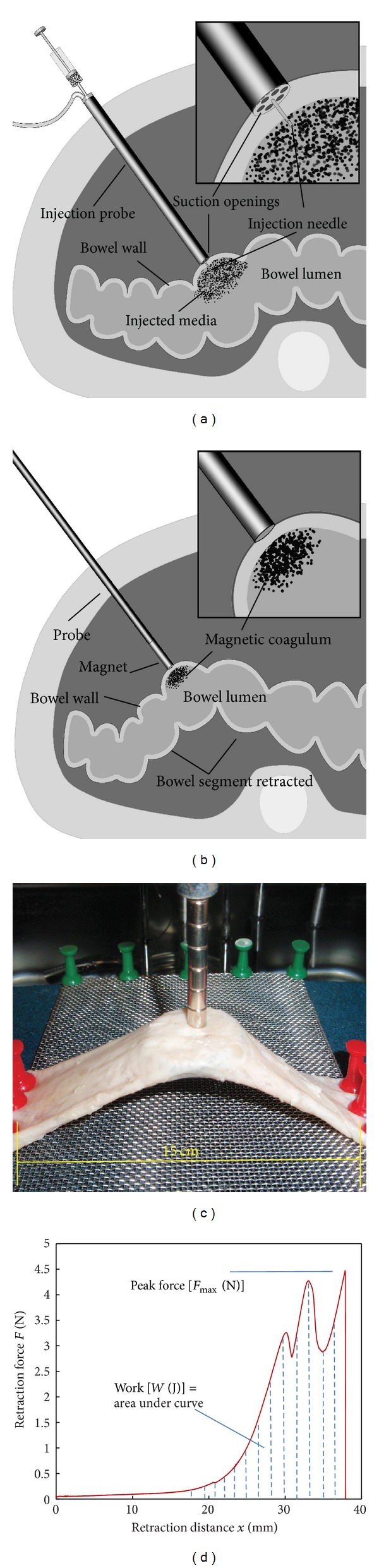
Magnetic retraction by intraluminal injection bowel magnetization. (a) Illustration of an intra-abdominal injection probe with suction openings for capturing bowel wall to facilitate intraluminal injection. (b) Illustration of an intra-abdominal magnet probe with distal magnet interacting with the injected magnetic glue attracted to the inner wall of the lumen by the abdominal magnet probe. (c) Photograph of an *ex vivo* porcine bowel experimental setup for magnetic bowel retraction force measurement. (d) Illustration of a recorded retraction force-distance curve for calculating peak force and work.

Figures 2(a) and 2(b) illustrates the components of the intraluminal retraction system for MAS procedure: (i) specially designed intra-abdominal injection probe was used with suction openings at its end to facilitate intraluminal injection of magnetic glue media into a bowel segment (Figure 2(a)); (ii) magnetic probe is held on the serosal side adjacent to the site of injection to ensure formation of a strongly magnetic coagulum, which adheres to the mucosal surface on polymerisation (Figure 2(b)); (iii) the magnetised bowel can then be retracted by the external magnetic probe.

We used neodymium iron boron (NdFeB) disc magnets with a remanence of 1.20 T (grade N35, Eclipse Magnetics Ltd., Sheffield, UK) of two diameters: 5 mm and 10 mm. Increasing the magnet's axial length can increase its magnetic field strength and attraction force to some degree, but we found that a magnet with a length/diameter ratio of 2.0 is optimal, as no significant force increase is achieved by longer magnets (data not shown). Additionally, a short distal magnet facilitates the design of both simple straight and complex custom-designed probes. 

The magnetic attraction force was measured using a tensiometer (Model 5564, Intron Ltd., Buckinghamshire, UK). Details on this measurement system have been reported previously [7, 8]. Briefly, the *ex vivo* bowel segment is fixed onto a support board with a free 15.0 cm segment (Figure 2(c)) and placed in a water bath at 37°C. After injection magnetisation, a magnetic probe connected to the tensiometer load cell was brought into contact with the bowel for retraction force measurement.  Figure 2(d) illustrates a recorded retraction force-distance curve. 

Several parameters can be derived from the recorded retraction force and distance curve (Figure 2(d)). Peak force [*F*_max⁡_ (N)] represents the maximal attraction (or bonding) force between the magnet and the magnetised tissue, and after reaching *F*_max⁡_, the probe starts to separate from the bowel until it detaches completely from it. The stress [*σ* (Pa)] at peak force is derived from dividing *F*_max⁡_ by the probe end-surface area. Work [*W* (J)] is defined as the area under the force-distance curve, which represents the work required for detachment of the two systems [23]. Work is calculated by a custom-written MATLAB (MathWorks, Cambridge, UK) program using (1) which is based on linear trapezoidal rule [24]:

(1) $$\begin{array}{c} W=\sum_{i=0}^{N-1}\left(x_{i+1}-x_{i}\right)*\frac{\left(F_{i}+F_{i+1}\right)}{2}, \end{array}$$

where *W* is adhesion work, *x* is retraction distance, *F* is retraction force, *i* is data sampling point, and *N* is total number of data points.

## 3. Results

### 3.1. Heat Generation by Glues

**Figure 3:**
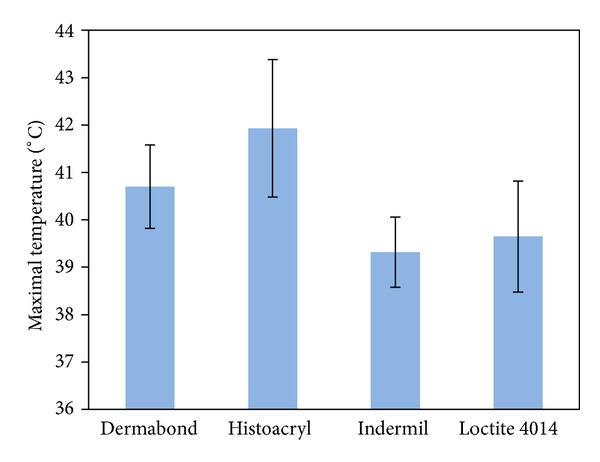
Averaged maximum temperatures were measured by the IR thermal camera after 0.5 mL of each glue was deposited onto the surface of *ex vivo* porcine bowel (error bar: standard deviation, number of test *n* = 7).

The maximal temperature generated during glue polymerisation by the 4 medical grade cyanoacrylate glues studied was obtained from the recorded thermal camera image.  Figure 3 plots the averaged maximal temperatures measured by the IR thermal camera after 0.5 mL of each glue was deposited onto the surface of *ex vivo* porcine bowel (error bar: standard deviation, number of test *n* = 7). All showed safe heat generation (i.e., below 42°C) except for Histoacryl (maximal about 43.5°C). 

### 3.2. Characterisation of Magnetic Particles

**Figure 4:**
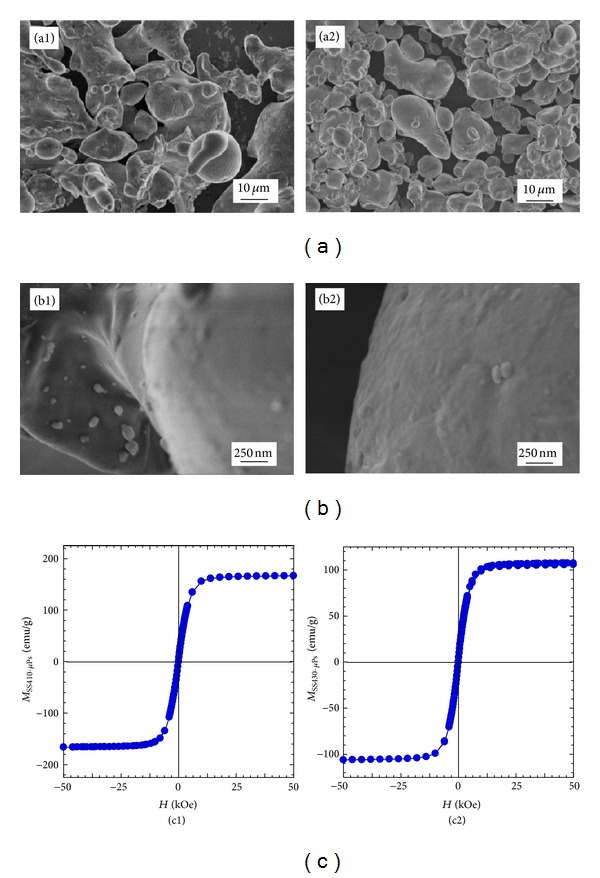
(a)-(b) SEM images and (c) hysteresis curves completed at room temperature for two stainless steel microparticle of SS410-*μ*Ps (1) and SS430-*μ*Ps (2).

Figures 4(a) and 4(b) present the appearance of stainless steel microparticles as observed by electron microscopy and Figure 4(c) the hysteresis curves at room temperature.

Results from XRD (not presented) and electron microscopy findings showed both SS410 and SS430 particles to be crystalline but polydispersed in size and shape with particles' diameters ranging from a few microns up to 50 *μ*m (Figures 4(a) and 4(b)). Magnetic properties in terms of magnetization as a function of applied magnetic field were quantified at room temperature and the resulting curves are shown in Figure 4(c). As expected the curves are symmetric and the magnetization at saturation, *M*_*s*_, is deduced from the plateau, when the magnetization does not increase any further with increasing magnetic field strength. SS410-*μ*Ps (Figure 4(c1)) exhibit a higher magnetization at saturation than SS430-*μ*Ps, and this is likely due to their slightly higher iron content and a lower chromium doping (Table 1). The coercivity refers to the magnetic field which needs to be applied to reduce the magnetization of a material down to zero after the magnetization of the sample has reached saturation. For both SS-*μ*Ps the remanence (i.e., the remaining magnetisation after the field has been removed) has been found to be very small. These observations indicate that both stainless steel *μ*Ps even though too large to form stable suspension would not present difficulty in dispersion in the fluid due to magnetic interactions since the particles exhibit no mutual magnetic attraction unless placed in a magnetic field. SS410-*μ*Ps were chosen for the study in view of their higher magnetization and lower coercivity (or remanence).

### 3.3. Measurement of Magnetic Bowel Retraction and Forces

Harvested porcine colonic segments were used with injection by a large gauge needle (16 G or 1.7 mm diameter × 50 mm, B. Braun Melsungen AG) to facilitate rapid injection of high concentration magnetic glue, although a smaller gauge needle (19G needle from BD Microlance 3, BD Drogheda, Ireland) can also be used for slower injection. After withdrawal of the needle, the small puncture wound effectively sealed itself by the injected glue with no visible leakage. The procedure took around 1 minute.  Table 2 summarises the magnetic retraction of a magnetised bowel segments in 20 *ex vivo* experiments using both the 5 mm and the 10 mm magnet probes. 

The average pull force that surgeons use to provide sufficient tension to the bowel is 2.5 N [1] with the maximal force being just below 5 N. The test results obtained in the present study (Table 2) indicate that SS410-*μ*Ps based bowel magnetization is capable of providing retraction forces and work in this range with use of 5 and 10 mm diameter permanent magnet probes. One important advantage of magnetic retraction over conventional pull traction by teeth-like graspers is that it inflicts less stress during high tension retraction with the tissues being not only retracted but compressed between the ridges of the graspers. For example, average pressures at the tip of conventional graspers at the contact surface area with the target tissue vary from 210 kPa to 650 kPa [25]. In contrast, the current approach does not require such compression and much lower pressures (65 kPa to 114 kPa) were observed in the current experiments by the 10 mm and 5 mm diameter magnetic probes, respectively (Table 2). 

### 3.4. On-Going Iron Oxide Nanoparticles Formulations

Magnetic nanoparticles can potentially exhibit stronger magnetic properties than microparticles per unit mass. This arises because bulk magnetic materials present multiple domains of magnetisations. Within each domain, the magnetisation has only one direction, which however can vary from one domain to another. The presence of several interacting domains in one particle with potentially different orientations can result in lowering the overall magnetisation of the particle. In contrast, decrease of the size of the particles to a single domain excludes the possibility of magnetic interactions between multiple magnetic domains within a single particle. If the nanoparticles do not carry a magnetic “dead-layer” at their interface, their magnetic properties can be enhanced compared to microparticles when exposed to the same magnetic field [26–28]. 

Magnetic nanoparticles have been successfully used for numerous biomedical applications [29–34]; however we found that iron oxide nPs could not be mixed easily with Loctite 4014 liquid for medical magnetic glue injection. For this reason the on-going studies by the group is exploring ways to overcome this problem as it is related to the fast polymerization of the glue when mixed with the iron oxide nanoparticles. This is likely associated with the surface reactivity of the nanoparticles which needs to be adequately tuned and possibly coated to enable development of efficient nanoparticle-based medical magneto-glues. 

## 4. Discussion

The new system based on formation of intraluminal magnetic coagulum provides highly effective atraumatic retraction and overcomes the problems reported with previous approaches based on localised ferromagnetisation of tissues, that is, low injection volume during interstitial ferromagnetisation [8] and peeling of surface magnetic pellets beyond certain retraction forces [7]. Furthermore, it provides significantly greater retraction forces, which meet all the requirements for uncompromised bowel retraction/manipulation during MAS equivalent to bowel grasping without risk of trauma or slippage. Additionally, the results of the present experiments have excluded thermal injury to the issues induced by medical grade cyanoacrylate glues during polymerisation. Loctite 4014 glue was selected because of its very low viscosity, single component nature, rapid cure rate, and ability to mix with stainless steel microparticles.

**Figure 5:**
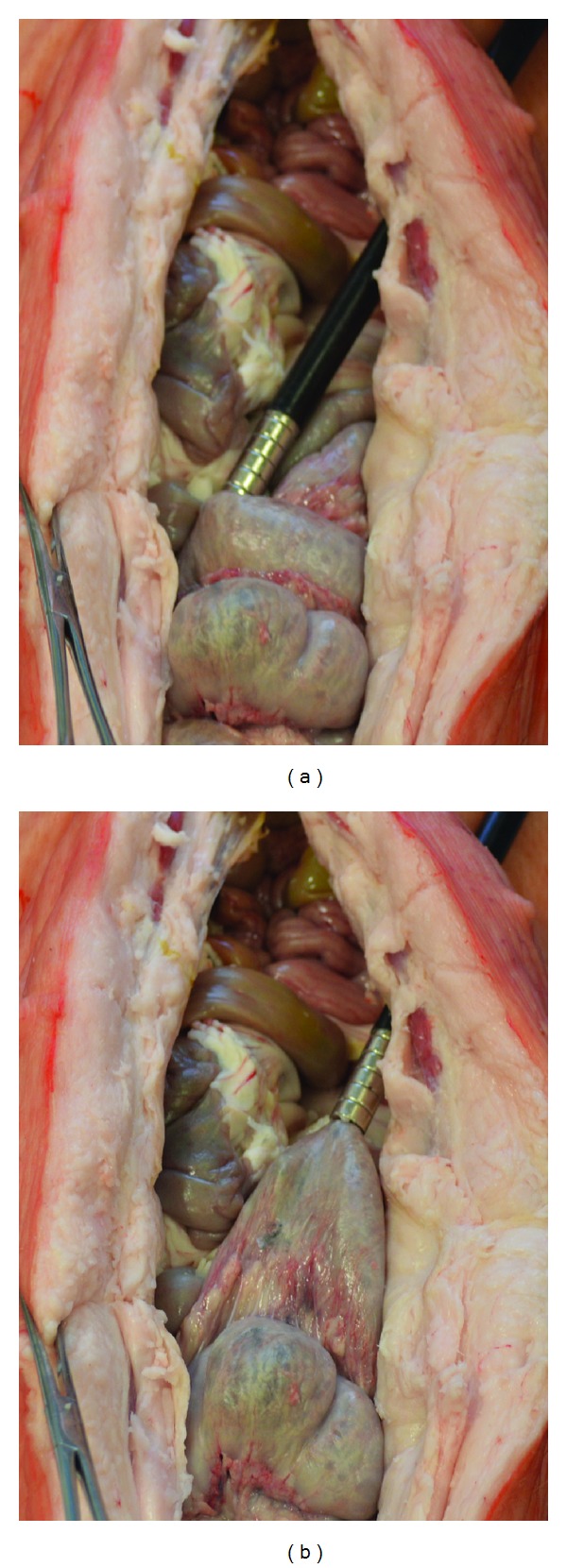
Retraction of in situ magnetised bowel using an *ex vivo* porcine model and a volume of 2 mL magnetic media with concentration of 1 g/mL was injected into a bowel segment: (a) photography showing the 10 mm magnet probe inserted into a 10 mm port and engaged with the magnetised bowel segment; (b) photography showing retraction of the magnetised bowel toward the port direction, with maximal detachment force of 5 N.

To date, *ex vivo* porcine bowel segments have been used to validate the concept and measure the forces generated (Figure 5). To facilitate intraluminal injection at randomly selected target bowel segment, an open abdominal model was used instead of a laparoscopic model. Two mL magnetic glue was injected intraluminally into targeted bowel segments and the 10 mm diameter magnet probe used to retract the magnetised bowel (Figure 5(a)) which could be manipulated and moved around, and retracted (Figure 5(b)) with maximal pull detachment force of 5 N, which was in agreement with Instron bench test results using *ex vivo* bowel segments (Table 2).

The *ex vivo* experiments with the formulated magnetic glue also demonstrated that even small volumes (2 mL) injected intraluminally in target bowel segments enable effective magnetic bowel retraction using a small (5 to 10 mm diameter) permanent magnet probes. For the same retraction force, the experimental data confirmed that the stress exerted on the target tissue by magnetic probes was significantly less when compared with the force generated during retraction by grasping forceps (65 kPa versus 210 kPa). Furthermore, the magnetic probe does not gather the tissue into a fold but instead creates a flat and smooth contact area with a wider distribution of probe-tissue contact forces. This underlies the atraumatic nature of magnetic retraction since the tissue falls off when the force exceeds the magnetic pull. Active release of the magnetically retracted tissue can be obtained by custom-designed probes which adjust the magnetic attraction force through a controlled release mechanism.

Our experiments also confirmed that change of retraction speed (from 0.1 mm/s to 10 mm/s) does not affect the magnetic retraction force and work, suggesting that bowel retracted with such probes would not have to be moved slowly during surgery. The experiments demonstrate that compared to external (serosal) application, the intraluminal magnetic coagulum significantly increases the maximal retraction force (1.75 ± 0.86 N versus 5.15 ± 0.98 N) with the use of a 10 mm magnet probe. Once the external magnet probe is removed, the coagulum detaches from the mucosa and thus becomes susceptible to spontaneous elimination in the stools. An adjustable magnetic force probe is desirable for controlled release of tissue whenever this is required. A simple and effective design for such a probe has been designed and developed in our lab and will be published elsewhere. 

## 5. Conclusions

The results of the current experimental study confirm that, with the technology described, intraluminal magnetic coagula formed from medical-grade biocompatible cyanoacrylate (Loctite) and SS410-*μ*Ps constitutes a novel system for efficient and atraumatic magnetic retraction of bowel. The system provides the retraction forces required for bowel manipulation and handling during laparoscopic surgery. The technology functions by producing a coagulum containing a sufficient mass of aggregated stainless steel microparticles adherent to the mucosa. This is either removed with the specimen in resectional bowel surgery or is expelled with return of bowel function. Further improvement is foreseen with the use of polymer coated nanoparticles of iron oxide being developed in our laboratory instead of stainless steel microparticles. Evaluation of the fully developed technology by *in vivo* large animal studies before translation to clinical practice is needed.

## Figures and Tables

**Table 1:** Characteristics of the magnetic particles.

	Size (diameter)	*M*_*s*_ (emu/g)	*H*_*c*_ (kOe)	Fe/Cr (w%)
SS410-*μ*Ps	Up to 50 *μ*m^SEM^	168.0	0.01	86.6 ± 6.8/12.6 ± 1.0
SS430-*μ*Ps	Up to 40 *μ*m^SEM^	110.0	0.02	82.2 ± 6.7/16.9 ± 1.4

Magnetisation at saturation (*M*_*s*_) and coercivity (*H*_*c*_) measured with a SQUID magnetometer at 300 K; iron and chromium percentage as deduced from ICP-OES measurements and size as obtained by SEM characterizations.

**Table 2:** Magnetic bowel retraction data.

	*F*_max⁡_ (N)	*W* (mJ)	*σ* (kPa)
5 mm magnet probe	2.2 ± 0.2	34 ± 11	114 ± 11.7
10 mm magnet probe	5.1 ± 0.3	81 ± 17	65 ± 4.3

SS410-*μ*Ps-based magnetic glues at concentration 1 g/mL: intraluminally injected volume of 2 mL.
